# Alzheimer’s Disease: Insights from Large-Scale Brain Dynamics Models

**DOI:** 10.3390/brainsci13081133

**Published:** 2023-07-28

**Authors:** Lan Yang, Jiayu Lu, Dandan Li, Jie Xiang, Ting Yan, Jie Sun, Bin Wang

**Affiliations:** 1 College of Computer Science and Technology, Taiyuan University of Technology, Taiyuan 030024, China; yanglan0085@link.tyut.edu.cn (L.Y.); lujiayu0413@link.tyut.edu.cn (J.L.); lidandan@tyut.edu.cn (D.L.); xiangjie@tyut.edu.cn (J.X.); sunjie0086@link.tyut.edu.cn (J.S.); 2 Teranslational Medicine Research Center, Shanxi Medical University, Taiyuan 030001, China; enntei@hotmail.com

**Keywords:** Alzheimer’s disease, large-scale brain dynamics models, alpha rhythm, neuronal excitability, abnormal regions of AD, neural mass model, Kuramoto model, brain network, neurodynamics

## Abstract

Alzheimer’s disease (AD) is a degenerative brain disease, and the condition is difficult to assess. In the past, numerous brain dynamics models have made remarkable contributions to neuroscience and the brain from the microcosmic to the macroscopic scale. Recently, large-scale brain dynamics models have been developed based on dual-driven multimodal neuroimaging data and neurodynamics theory. These models bridge the gap between anatomical structure and functional dynamics and have played an important role in assisting the understanding of the brain mechanism. Large-scale brain dynamics have been widely used to explain how macroscale neuroimaging biomarkers emerge from potential neuronal population level disturbances associated with AD. In this review, we describe this emerging approach to studying AD that utilizes a biophysically large-scale brain dynamics model. In particular, we focus on the application of the model to AD and discuss important directions for the future development and analysis of AD models. This will facilitate the development of virtual brain models in the field of AD diagnosis and treatment and add new opportunities for advancing clinical neuroscience.

## 1. Introduction

Alzheimer’s disease (AD) is a brain disorder characterized by the accumulation of amyloid-based neuritic plaques and the abnormalities of neurodegeneration [1]. In particular, there is a progressive loss of neurons or their processes (axons and dendrites), and the corresponding neuronal function is impaired [2]. Similarly, AD is affected by age, biological sex, and other related factors. Progressive cognitive and behavioral impairment will be presented at different stages of AD (early, moderate, and severe phases) [3,4].

In recent years, substantial advancements have been made in the diagnosis, treatment, and prevention of AD via systematic implementation based on multimodal neuroimaging modalities, cognitive assessment tests, and clinical characteristics [5]. At the microscale level, many different alterations may eventually lead to synaptic decay [6,7]. For example, microcircuit dysfunction in the brain of AD patients is associated with the synaptic inhibition of excitatory neurons [8]. It has also been shown that changes in the morphology and density of dendritic spines have been postulated as the main reasons for the synaptic and neuronal loss observed in AD [9]. Additionally, hyperphosphorylated τ deposition and amyloid-β (Aβ) both impair neuronal functions and ultimately lead to axonal degeneration [4]. At a vastly different level of analysis, the macroscopic level, non-invasive neuroimaging has been used as a vector for diagnostic biomarkers of AD due to its excellent speed, considerable spatial resolution, and ability to record neuronal activity [10,11,12], and it also plays a crucial role in the clinical identification of MCI and AD [13,14]. It is understood that deficiencies caused by AD will affect cognitive functions in large-scale brain networks, but how it will do so is not yet clear. Even more important for AD, traditional non-invasive methods capturing brain activity for early changes have not yet yielded specific, generally accepted features of underlying neuronal hyperactivity [15,16,17], and newer approaches based on source localization or non-invasive brain stimulation still require further validation [10,11,18].

Large-scale brain dynamics models based on nonlinear dynamical systems allow for integrating experimental data from multiple modalities into a common framework [19] and help us to better understand the neurophysiological changes in AD. According to existing research findings, models can reveal the structural and functional relationships of the brain [20,21,22], understand the working mechanisms of the brain [23,24], explore the pathogenic mechanisms of brain diseases [25,26,27], and explain processes such as the development or evolution of the brain neural system [28,29]. Given that AD is a complex multifactorial disease characterized by neuronal degeneration and neuronal synaptic disorders in cortical and subcortical areas, as well as abnormalities of white and gray matter, it is better suited to be studied with large-scale brain dynamics models. Using neurodynamic model simulations, researchers have demonstrated that the disconnection of macroscopic anatomical structures in the brains of AD patients leads to abnormal changes in metastability, and this revealed an important relationship between metastability, cognition, and the efficiency of anatomical structures [30,31]. In addition, other researchers have used models that can analyze the characteristics of the evolutionary process to identify changes in alpha rhythms [32,33] and excitatory neurons [6,34] in the brains of AD patients and areas of abnormality [31,35,36] between AD patients and controls.

In this review, we systematically examined 24 identified studies on the application of large-scale brain dynamics models in AD from 2010 to 2023. We aim to review the large-scale brain dynamics models that are available for AD research and summarize the findings from studies using these models. In addition, we also highlight opportunities that will require the further development of models about AD (Table 1). In particular, we emphasize the importance of incorporating more information in the model as well as in the modeling process and in the selection of longitudinal data. This also presents a key challenge to adequately constrain neuroimaging data and construct models with these relevant properties. In summary, large-scale brain dynamics models can provide new opportunities for advancing clinical neuroscience.

## 2. Materials and Methods

In this paper, we investigate the application of large-scale brain dynamics models in AD. Preferred Reporting Items for Systematic Reviews and Meta-Analyses (PRISMA) [49] was used to identify studies and narrow the collection for this review. This study was registered at PROSPERO. We performed a search on PubMed and Web of Science using the following group of keywords: “Alzheimer’s Disease” AND (“Neural dynamics” OR “Brain dynamics” OR “neuroanatomy” OR “dynamical systems” OR “Nonlinear dynamics”) AND (“Computational model” OR “Whole brain computational model” OR “computational modeling” OR “mathematical model” OR “Neural activity”) AND (“large-scale” OR “large scale” OR “macroscale”).

**Figure 1:**
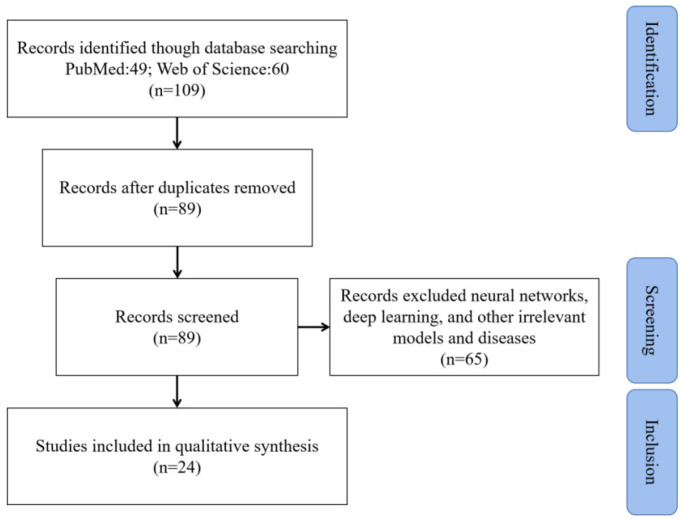
Selection diagram, including three stages: identification, screening, and inclusion. This process led from 109 initial studies to 24 final studies.

References from 2010 to 2023 were used for further analysis. As shown in Figure 1, after excluding unqualified studies, this review narrowed the original count of 109 studies to the final count of 24 studies.

## 3. Large-Scale Brain Dynamics Models

### 3.1. Neural Dynamics Models

In the field of neuroscience, theory and computational models are playing increasingly important roles in the challenges of understanding complex biological dynamic systems. The neurodynamic models constructed based on real collected imaging data can not only discover some phenomenon that conforms to statistical significance but also adequately explain mechanistically and conduct systematic studies on the development, evolution, or dynamics of the brain nervous system [19]. Currently, models play an important role in the “observation-experiment-theory” phase of comprehensive exploration, especially on the basis of the pioneering work of Hodgkin and Huxley [50]. As the model evolved, the neural mass model was formed in order to bridge the gap between the activity of individual neurons and the circuits [51,52]. This was later followed by the construction of the continuum neural field model [53], which can be extended in space, and the whole-brain network model that can be applied clinically [52,54].

**Figure 2:**
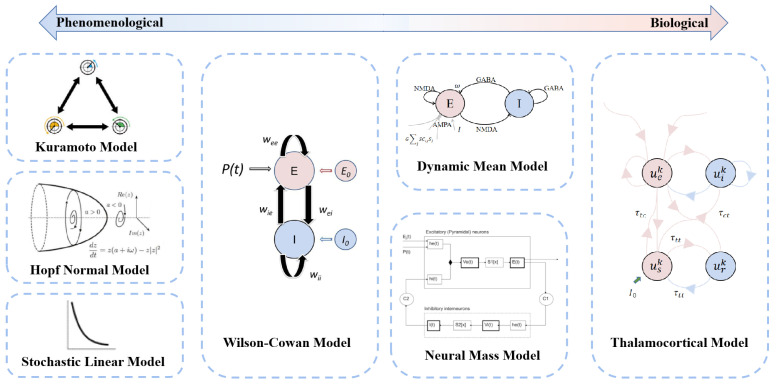
Classification of neurodynamic models on the biological information axis.

Neural dynamics models simulate brain dynamics using biologically inspired mathematical equations that model regional activity and are coupled through the observed brain structure [21]. These equations can be built from knowledge concerning the biophysical mechanisms underlying different forms of brain activity or as phenomenological models chosen by the kind of dynamics they produce [55]. Thus, the models are also classified into two main categories: biologically informed models and phenomenologically informed models [27]. Depending on the amount of biological information covered in the models, the models are represented on the biological information axis in the position of the category to which it roughly belongs (Figure 2). In the context of phenomenologically informed models, we tend to consider more the phase transition for global connectivity and ignore the details of the model [26,43]. For example, the Kuramoto model, used to study network synchrony, considers time-delayed interactions between nodes [56,57]; the Hopf normal model has the advantage of allowing transitions between asynchronous noise activity and oscillations [35,58]; and the stochastic linear model is a linear stochastic system of coupled Ornstein–Uhlenbeck processes [59]. In the context of biologically informed models, we could treat the brain as a large set of single-cell neurons that cover the entire brain and are wired using white matter tract information from diffusion tensor imaging (DTI) [27]. For instance, a number of researchers have used the neural mass model, which describes a local population of interacting neurons, such as pyramidal and inhibitory cells [19]. This model is constructed in two ways: one uses the same conductance-based model as a single-neuron model to model the mean ensemble activity; the other uses careful empirical observations to understand and model the response of the system to inputs, as is the case with the Jansen–Rit model [19]. An additional class of models, dynamic mean-field models, has been used, which includes some of the more refined local models with an ability to model the balance between excitation and inhibition. These models are based on firing rates and synaptic gating activity [40,60]. There is also the Wilson–Cowan model [51], which uses a phase-plane approach and numerical solutions to describe the response of neuronal populations to stimulation, and the thalamocortical model [61], which describes the interaction of cortical and thalamic populations using a delayed feedback loop. A detailed review of the different models can be found in [52,62].

### 3.2. Large-Scale Dynamics Model Building

**Figure 3:**
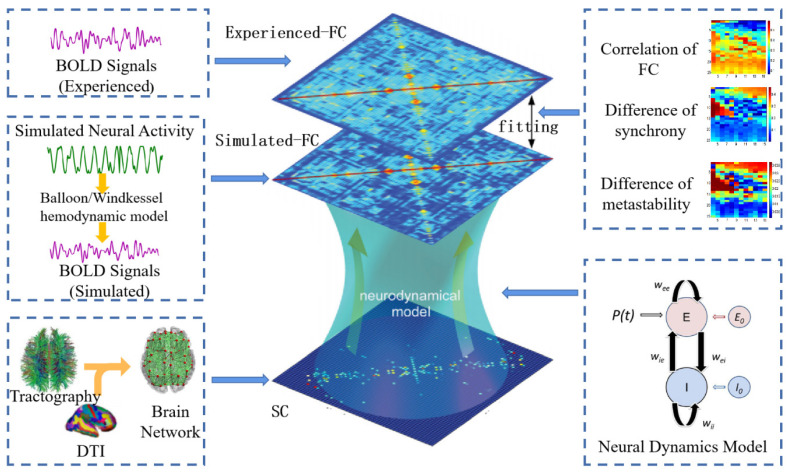
Modeling process of large-scale brain dynamics models. The multimodal neuroimages are preprocessed to extract the empirical SC and FC. Then, a large-scale brain dynamics model with SC coupling constraints is used to simulate neuronal activity in the brain, converting the simulated neuronal activity into BOLD signals and extracting the FC. Finally, the empirical and simulated FC, synchrony, and metastability are fitted to find and determine the optimal parameters of the model to simulate the closest functional activity of the real brain.

The study of model building can be divided into three parts: microscopic, mesoscopic, and macroscopic. The interrelationships between network structure and dynamics are largely elusive at the micro- and mesoscopic level [63]. From a purely neuroscientific perspective, modeling studies are important to provide fundamental insight into the driving factors of connectivity fluctuations, which will potentially bridge the gap between microscopic and macroscopic activity and connectivity [27]. The model architecture requires two key elements: (1) a structural connectivity matrix defining the coupling between areas, which can be derived from empirical data; (2) equations governing the neural dynamics of each local node, which can be constrained by known neurophysiology [64,65]. The specific modeling process is shown in Figure 3.

Structural connectivity, as an essential part of the modeling process, specifies fiber density across various white matter tracts [52]. Together with the brain parcellation that determines the number of regions and the spatial resolution where brain dynamics take place [55], large networks are constructed. In models, elements of the structural connectivity matrix set the strength of the coupling between nodes [65]. Also, in previous studies, the choice of parcellation was found to be important. For example, subcortical nodes are also crucial where the main pathology may involve subcortical structures [52] such as the hippocampus [66], the thalamus [41], and so on.

Functional connectivity is defined as the temporal dependence of the neuronal activity patterns of anatomically separated brain regions and is typically measured as the co-variation between brain signals originating at different locations [62]. This has been particularly useful for the optimization and validation of models via the comparison with the FC matrix obtained using model simulations [56,62,67].

Given network dynamics evident spontaneous emergence and their spatial similarity with the underlying structural connectivity, whole-brain network models are useful tools for investigating the biophysical mechanisms underlying brain activity [62]. By considering the dynamics emerging spontaneously from the interplay between brain areas when these are embedded in the neuroanatomical network, one can analyze the observed phenomena in the light of empirical data and formulate scenarios for the physiological origin of brain activity [62]. And a number of theoretical works have used reduced whole-brain network models to investigate the activity emerging from the interplay of brain areas in the neuroanatomical network [62,67,68]. The activity of each brain area is represented by one or more mathematical expressions representing the spontaneous behavior of isolated brain areas with an additive term representing the input received by anatomically connected areas [62].

Another point of the modeling process that needs attention is model fitting. The model parameters can be calibrated in such a way that the model dynamics closely replicate the dynamics of the brain networks extracted from the empirical data [20]. Most previous studies have used static correlations to estimate the model fit. However, as the research advanced, the use of dynamical metrics, such as functional dynamic connectivity [62], synchrony, and metastability [67], was proposed to further constrain the model fitting in order to preserve the dynamic time characteristics present in the signals at different functional states. These make use of exploring the parameter space to find the optimal parameter set by minimizing the error function or the maximizing correlation between empirical and simulated matrixes, but currently there are also iterative-fitting strategies used to update the model parameters [26,69].

Intuitively, the potential utility of brain network dynamics models is large, and it is not only influenced by biological mechanisms but also closely related to whole-brain neuroimaging data. From the summary of existing studies, it is clear that from the perspective of acquisition, the results of brain network dynamics model simulation can solve the problems of difficult data acquisition to a certain extent, such as the small number of patients with brain diseases and the inability of patients to remain quiet during acquisition. The model can also simulate data at different resolutions, allowing for a more detailed study of the content in question. In addition, the simulation results can avoid the influence of noise during the acquisition process, thus obtaining data that more closely resemble the real brain. More importantly, depending on the changes in the real data set, some of the study findings are not reproducible, but the results simulated using the model avoid such effects. From the perspective of applied research, brain network dynamics models are more able to reflect the principles of the biophysical mechanisms of the brain because they can reflect the relationship between brain structure and function. While human brains cannot be used for damage or stimulation studies due to ethical concerns, researchers can construct a personalized brain network dynamics model by changing the collected structural data so that they can be studied in greater depth. The researcher can construct a personalized brain network dynamics model by altering the collected structural data to study the data at a deeper level. The brain network dynamics model can predict the subsequent state from the existing state of the brain, thus providing a beneficial tool for the evolutionary process of brain aging and disease prevention and treatment. At the same time, this provides new opportunities to advance clinical neuroscience.

## 4. Applications of Large-Scale Brain Dynamics Models to Alzheimer’s Disease

Here, we focus on applications to AD (Table 1). As a neurodegenerative disease, AD is a disease that integrates the pathogenesis of excitatory synaptic deficits, slowed oscillatory rhythms, and regional changes. Most of the current modeling studies have been analyzed from these three aspects.

### 4.1. Slowing of Alpha Rhythm: A Biomarker for AD

Each oscillatory frequency of brain activity conveys specific physiological information on brain functional states during sleep and wake periods. The alpha rhythm represents memory and attentional processes, which functionally inhibit task-irrelevant brain regions and transmit information to task-relevant regions [70]. Low-frequency oscillations are associated with functional inhibition, so the slowing of the alpha rhythm is thought to better characterize AD [41]. Initially, Pons et al. found a slowing of the alpha rhythm and an increase in functional connectivity in older MCI patients based on a neural mass model [71]. Subsequently, a more detailed study by van Nifterick et al. found that a relatively higher theta power and a lower beta power followed by a decrease in alpha power at later stages could serve as a mechanism for well-established AD hallmarks [44]. The same conclusion was demonstrated by Ruiz-Gomez et al. using the Kuramoto model [33]. Meanwhile, current researchers have improved the thalamo-cortico-thalamic (TCT) model and suggest that this model is relevant to AD and that it is a good candidate for understanding the thalamo-cortico-thalamic neuronal mechanisms associated with alpha rhythm slowing observed in AD [32]. Based on these studies, a point was proposed: the decrease in synaptic strength has a dominant effect on the slowing of the alpha rhythm [32,34,41]. This established the basis for the following studies.

### 4.2. Altering Neuronal Excitability: A Therapeutic Strategy for AD

The impairment of cognitive function in Alzheimer’s disease is clearly correlated to synapse loss [6]. Using a spiking neural network model, Bachmann et al. found that the loss of excitatory synapses drives the network into a less sensitive regime, which may also account for the cognitive deficits of AD [6]. To study the potential etiology of AD and simulate the Abeta-induced disruption of interneuronal inhibitory function and consecutive hyperexcitability, Stefanovski et al. proposed a mathematical model that adds a description of molecular changes to alter population activity, where individual Abeta burden modulates regional excitation–inhibition balance, leading to local hyperexcitability with a high Abeta load in personalized virtual brains [34]. This also builds a bridge between molecular pathways and brain network modeling.

Similarly, de Haan et al. used a large-scale neural mass network to simulate the temporal evolution of activity-dependent network degeneration, and the results showed that functional network damage can be opposed by the targeted adjustment of neuronal excitability levels [39]. Van Nifterick et al. combined model and human data and found that neuronal hyperactivity may be due to hyperexcitation and/or disinhibition in early AD [44]. Therefore, we can consider increasing the excitability of excitatory neurons, followed by increasing the inhibition of inhibitory neurons as current effective therapeutic strategies.

### 4.3. Abnormal Regions of the Brain: Potential Therapeutic Target Areas

AD is characterized by the degradation of the connectome and of network organization by these long-range connections, with both structural and functional connectivity being affected [40]. It has been proposed that damage to the connectivity of an AD computer model focuses on nodes with high topological centrality, integration, and efficiency scores and builds connections between structure and cognition via metastability [31]. This also provides a unique perspective for AD research.

In the existing study, the relation between functional connectivity and complexity was explored using the Kuramoto mean-field model in the MCI-AD condition, and certain variations were found in the anterior and central regions in the results, specifically including the frontal and temporal lobes [36]. And in Zimmermann et al.’s study, the researchers attempted to identify biophysical model neural parameters that were associated with cognition along the spectrum from healthy controls to mild cognitive impairment (MCI) to AD, modeled the limbic subnetwork as well as the entire brain, and found that neurodegeneration and functional changes occurred first in the limbic and temporal regions of the brain and, later, in the motor and sensory areas [40].

Demirtas et al. proposed a whole-brain computational model to explore the mechanisms behind changes in whole-brain and regional connectivity and, among the results, found that regions of FC impairments in AD overlap with the default mode network (DMN) and that decreased effective connectivity (EC) strength primarily affects the left temporal lobe, instead of prompting widespread decreases in FC strength [35]. It was also found that preclinical Alzheimer’s disease (PAD) subjects exhibited decreased EC strength in several regions including the left hippocampus, while no significant EC differences were found in the MCI group [35]. This has been interpreted according to previous studies as a compensation mechanism for maintaining cognitive function under pathology-related insults in the preclinical stage of AD [72,73]. It is worth noting that Bachmann also proposes a circuit compensation mechanism for AD treatment, which restores the mean network activity by increasing the weight of the remaining connections [6]. Now, the limbic system and basal ganglia structures constitute the primary target locations for stimulation in AD [74].

## 5. Future Directions

Large-scale brain dynamics models of AD have been presented as useful tools to fill the gap between theoretical models and biological evidence. They link microscopic excitatory–inhibitory neurons, mesoscopic fiber tract connections, and macroscopic brain function and cognition, providing new ideas for exploring AD pathogenesis. In addition, the model-based optimization of neuromodulation schemes can be performed without ethical constraints. By giving stimulation to the AD-simulated brain and adjusting it according to the post-stimulation neural activity, it is possible to predict whether the stimulation protocol can normalize the abnormal network dynamics characteristics of AD and give an interpretable neuromodulation strategy. However, the challenges associated with the integration of findings from different levels of analysis (micro-, meso-, and macroscale) are delaying the development of models of the disease [18]. Thus, we have suggested some areas that could be further optimized.

At present, most researchers analyzing the mechanisms of brain activity from a microscopic and mesoscopic perspective have found that these influencing factors play an important role. However, large-scale brain dynamics models only focus on valid information about whole brains or the relationships between local regions, ignoring factors at a more detailed scale. In this sense, the model should be considered to include more information on biological details. In particular, in AD, degenerative changes in several neurobiological processes spanning molecular to macroscopic scales are involved, including proteinopathies, modified gene expression, synaptic alterations, vascular dysregulation, hypometabolism, and structural atrophy [75,76]. Identifying important pathways between truly microscopic-scale variables and observable macroscopic neuroimaging in Alzheimer’s disease would both advance the understanding of the underlying biology and improve the selection of therapeutic targets tailored to an individual’s particular disease subtype or presentation [76]. Therefore, we should incorporate more biological features into the model for the more accurate simulation of brain dynamics.

The investigation of the strength and directionality of interactions in the resting human brain could help to better understand the cognitive deficits associated with network disruption in certain neurological disorders and provide new biomarkers of these disorders [77]. Directed FC between the hippocampus and other brain regions had significant differences among NC, MCI, and AD subjects, and these differences were significantly correlated with cognitive and clinical measurements [78]. EC has also been proposed and has been demonstrated to reveal the direction of information flow between different brain regions, providing new insights into the underlying neuronal alterations in MCI and AD [79]. However, at present, we have not considered the directionality of the connection network when building the model, and this is one of the key elements to be considered for optimization.

We also found that only a few features of brain nodes have been focused on in some studies, and the attributes are more singular. For example, Tijms et al. used cortical thickness as a nodal attribute and constructed a gray matter brain network by calculating Pearson similarity. They found that MCI patients had lower values of standardized clustering coefficients and small-world attributes, which would become even lower as the disease progressed [80]. In contrast, the brain attribute network [81] contains nodal attributes and connectivity information, which can describe the structure and function of the brain from multiple perspectives and better characterize the subtle changes in the brains of patients with different stages of AD. If we construct a model using the brain attribute network, this will provide more advantages in studying the pathogenesis and progression of AD.

In addition, to date, studies of brain networks in AD have been mainly cross-sectional. In contrast, using longitudinal data from the whole brain, information from two points in time can be brought together simultaneously to generate different idiosyncratic networks for AD, resulting in more accurate inferences [82]. Moreover, the models can be used to analyze the characteristics of the disease evolution process and understand the progression of AD using longitudinal data, which is also important for developing treatment plans and predicting the trend of disease progression. Therefore, subsequent studies may also consider using longitudinal data for analysis.

## 6. Conclusions

In this review, we have shown that large-scale brain dynamics models can accurately explore and characterize brain mechanisms in AD patients. We provide a brief overview of the neural dynamics model and its large-scale modeling process. The application of models to AD concerning alpha rhythms, excitatory neurons, and abnormal regions is also summarized and discussed. Like other fields, the building of large-scale brain dynamics models for AD needs further development to realize its greater potential, and we provide methodological and technical recommendations in terms of the models themselves, the modeling process, and data selection. In summary, biophysically based whole-brain dynamics models are well positioned to impact the treatment field of AD. This also sets the stage for ushering in personalized medicine.

## Figures and Tables

**Table 1:** List of studies employing large-scale brain dynamics models to understand AD.

References	Dynamics Model	Subject	Imaging Modality	Means of Simulating AD	Main Conclusion
(de Haan et al. 2012) [37]	Neural Mass Model	NC\MCI\AD	EEG	Lowering synaptic strength	Excessive neuronal activity and hub vulnerability.
(Ghorbanian et al. 2014) [38]	Coupled Duffing–van der Pol Oscillator Model	NC\AD	EEG	Simulation based on empirical data	α dominant for CTL subject and θ dominant for AD patients.
(de Haan et al. 2017) [39]	Neural Mass Model	NC\AD	EEG	Changing neuronal excitability levels due to varied threshold potential (Vd) settings	AD-like network degeneration can be countered by global stimulation of excitatory neurons.
(Demirtas et al. 2017) [35]	Hopf Normal Model	NC\PAD\MCI\AD	fMRI	Manipulating the bifurcation parameter	Synchronization ↓, FC strength ↓, and the significant EC differences in AD were located in left temporal lobe.
(Zimmermann et al. 2018) [40]	Reduced Wong–Wang Model	NC\MCI\AD	fMRI	Simulation based on empirical data	The model parameters correlated with cognition and the predictive capacity ↑.
(Alderson et al. 2018) [31]	Kuramoto Model	NC\MCI\AD	fMRI	Lesioned the structural connections of healthy subjects	Metastability ↓; damage was centered around highly connected nodes; abnormal network topology; a link between metastable neural dynamics, cognition, and the structural integrity of the human brain.
(Stefanovski et al. 2019) [34]	Jansen–Rit Model	NC\MCI\AD	fMRI	Based on individual PET-derived distributions of Abeta	Spatial heterogeneous Abeta distribution, impaired inhibitory function, and neural frequencies ↓.
(Ruiz-Gomez et al. 2019) [33]	Kuramoto Model	NC\MCI\AD	EEG	Simulation based on empirical data	Θ band ↑ and α band ↓.
(Cakir 2020) [41]	Izhikevich Model and Neural Mass Model	NC\AD	fMRI	Simulation based on empirical data	The alpha rhythms ↓ in the thalamic, and fALFF of slow-4 band ↓ in the striatum.
(Li et al. 2020) [32]	Thalamo-cortico-thalamic (TCT) Circuitry Model	Simulated AD	EEG	Decreasing synaptic connectivity parameters	Synapse loss and alpha rhythm ↓.
(Bachmann et al. 2020) [6]	Leaky Integrate-and-Fire (LIF) Model	Simulated AD	EEG	Adjusting the weight of excitatory synapses	The loss of excitatory synapses on excitatory neurons.
(Arbabyazd et al. 2021) [42]	Stochastic Linear Model (SLM) and Mean-Field Model (MFM)	NC\MCI\AD	fMRI	Simulation based on empirical data	Realistic data can be generated by whole-brain modeling.
(Triebkorn et al. 2022) [43]	Jansen–Rit Neural Mass Model	NC\MCI\AD	fMRI	Adjusting the global scaling factor G and linked local Aβ concentrations	Local hyperexcitation caused by Aβ can classify AD.
(van Nifterick et al. 2022) [44]	Neural Mass Model	SCD\MCI\AD	MEG	Adjusting single relevant parameters	Oscillatory ↓; hyperexcitation.
(Das and Puthankattil 2022) [36]	Kuramoto Model	NC\MCI-AD	EEG	Edges originating from one specific region of the cortex are set to the lowest value	Functional connectivity ↓ and complexity ↓ in anterior and central regions.
(Patow et al. 2023) [45]	Balanced Excitation–Inhibition Model	NC\MCI\AD	fMRI	Adjusting the inhibitory bias and scaling parameters	The neuronal activity of Aβ over tau in MCI, while tau dominates over Aβ in AD.
(Salimi-Nezhad et al. 2023) [46]	Pinsky–Rinzel Neuron Model	Healthy rats	LFP	Eliminated 75% of MS cholinergic neurons	Selectively stimulating the remaining healthy cholinergic neurons was sufficient for network recovery.
(Sanz et al. 2023) [47]	Hopf Normal Model	NC\AD\bvFTD	fMRI	Simulation based on empirical data	Key nodes to transition from AD towards the healthy state included the hippocampus as well as temporo-posterior regions.
(Alexandersen et al. 2023) [48]	Neural Mass Model	NC	E/MEG	Adjusting the Aβ and τP damage variables	Excitatory neuronal activity↓; oscillatory↓ independently of structural changes due to axonal damage.
